# Optimizing the Measurement of Information on the Context of Alcohol Consumption Within the Drink Less App Among People Drinking at Increasing and Higher Risk Levels: Mixed-Methods Usability Study

**DOI:** 10.2196/50131

**Published:** 2024-10-24

**Authors:** Abigail K Stevely, Claire Garnett, John Holmes, Andrew Jones, Larisa Dinu, Melissa Oldham

**Affiliations:** 1 Sheffield Addictions Research Group School of Medicine and Population Health University of Sheffield Sheffield United Kingdom; 2 Research Department of Behavioural Science and Health University College London London United Kingdom; 3 School of Psychological Science University of Bristol Bristol United Kingdom; 4 School of Psychology Liverpool John Moores University Liverpool United Kingdom

**Keywords:** alcohol use disorder, substance use disorder, alcohol consumption, mobile app, mHealth, mobile health, diary, health behavior change, usability, user engagement

## Abstract

**Background:**

There is a growing public health evidence base focused on understanding the links between drinking contexts and alcohol consumption. However, the potential value of developing context-based interventions to help people drinking at increasing and higher risk levels to cut down remains underexplored. Digital interventions, such as apps, offer significant potential for delivering context-based interventions as they can collect contextual information and flexibly deliver personalized interventions while addressing barriers associated with face-to-face interventions, such as time constraints.

**Objective:**

This early phase study aimed to identify the best method for collecting information on the contexts of alcohol consumption among users of an alcohol reduction app by comparing 2 alternative drinking diaries in terms of user engagement, data quality, usability, and acceptability.

**Methods:**

Participants were recruited using the online platform Prolific and were randomly assigned to use 1 of the 2 adapted versions of the Drink Less app for 14 days. *Tags* (n=31) included tags for location, motivation, and company that participants added to drink records. *Occasion type* (n=31) included a list of occasion types that participants selected from when adding drink records. We assessed engagement and data quality with app data, usability with a validated questionnaire, and acceptability with semistructured interviews.

**Results:**

Quantitative findings on engagement, data quality, and app usability were good overall, with participants using the app on most days (*tags*: mean 12.23, SD 2.46 days; *occasion type*: mean 12.39, SD 2.12 days). However, around 40% of drinking records in *tags* did not include company and motivation tags. Mean usability scores were similar across app versions (*tags*: mean 72.39, SD 8.10; *occasion type*: mean 74.23, SD 6.76). Qualitative analysis found that both versions were acceptable to users and were relevant to their drinking occasions, and participants reported increased awareness of their drinking contexts. Several participants reported that the diary helped them to reduce alcohol consumption in some contexts (eg, home or lone drinking) more than others (eg, social drinking) and suggested that they felt less negative affect recording social drinking contexts out of their home. Participants also suggested the inclusion of “work drinks” in both versions and “habit” as a motivation in the *tags* version.

**Conclusions:**

There was no clearly better method for collecting data on alcohol consumption as both methods had good user engagement, usability, acceptability, and data quality. Participants recorded sufficient data on their drinking contexts to suggest that an adapted version of Drink Less could be used as the basis for context-specific interventions. The *occasion type* version may be preferable owing to lower participant burden. A more general consideration is to ensure that context-specific interventions are designed to minimize the risk of unintended positive reinforcement of drinking occasions that are seen as sociable by users.

## Introduction

In 2019, the global mortality from alcohol consumption was estimated to be 2.44 million [1], and alcohol consumption was the 9th leading risk factor for disability-adjusted life years [1]. These harms are particularly experienced by people drinking at increasing and higher risk levels, typically defined in the United Kingdom as drinking above the low-risk guidelines for alcohol consumption [2]. Traditionally, interventions to reduce alcohol consumption and subsequent alcohol-related harms focus on individual-level risk factors and psychological traits [3,4]. However, there is increasing theoretical and empirical support for considering the contexts in which drinking occurs, such as the location (eg, drinking in pubs or at home) or the company (eg, drinking with a large group of friends or alone) [3-7]. A recent study found that between 55% and 71% of the variance in alcohol consumption was explained by the context of drinking occasions, such as occasion duration, beverage type, or having an informal meal [7], and analyses of occasion-level data suggested that alcohol consumption and heavy drinking are more prevalent in some contexts than others [8,9]. These findings suggest that developing effective context-based interventions may be beneficial to the individual, as well as wider public health. The aim of this study was to develop an existing digital intervention, *Drink Less*, to measure the context of drinking occasions.

Public health research in this field has drawn on a range of disciplinary and theoretical perspectives [5]. Our work has applied sociological theories of practice, which conceptualize alcohol consumption as occurring within a set of recognizable “practices” that are routinely performed as part of people’s daily lives [3,7,9,10]. For example, the practice of “a big night out” or “going to the pub with male friends” [9]. However, the implications of a practice theoretical perspective for developing interventions to help people drinking at increasing and higher risk levels to cut down remain underexplored. Practices that involve drinking may be more or less amenable to change since, for instance, the importance or irreplaceability of heavy drinking within the practice may vary. Similarly, Meier et al [3] argued that the effectiveness of intervention strategies is likely to vary across practices.

Digital interventions, such as apps, offer significant potential for delivering context-based interventions as they can collect contextual information and flexibly deliver personalized interventions while addressing barriers associated with face-to-face interventions, such as time constraints or lack of confidence among health professionals [11]. However, we are not aware of any alcohol reduction apps to date, which have measured drinking contexts or provided targeted advice. Some smoking cessation apps, such as the SmokeFree app [12], allow users to track routines and triggers to smoking, which could involve certain contextual factors such as being in a pub or with a coffee in the morning. However, smoking is different in that each smoking “occasion” is shorter, and for many smokers, smoking occurs constantly throughout the day in many different contexts.

The Drink Less app is an existing theory- and evidence-informed alcohol reduction app developed by the authors and colleagues [13,14]. The app includes a diary where users enter the alcoholic drinks that they had each day (or a drink-free day), as well as intervention components, such as goal setting, where users can enter and track goals (eg, having 3 alcohol-free days a week) [14]. A large-scale randomized controlled trial funded by the National Institute for Health and Care Research (NIHR) found that the Drink Less app reduced alcohol consumption compared with usual digital care and was cost-saving [15]. This study is part of a wider project aiming to improve the efficacy of the Drink Less app by recording contextual information on users’ drinking occasions and using this within context-specific intervention components.

The pre-existing drinking diary in the Drink Less app does not record any information about the context of users’ drinking occasions. We developed 2 potential methods to measure self-reported drinking contexts within the diary. First, users could record multiple separate characteristics of drinking occasions (ie, location, company, and motivation) in the drinking diary by selecting tags when recording each drinking occasion (*tags*). This approach offers users the flexibility to record any combination of tags to describe many different types of drinking occasions, but it may be burdensome to enter this level of detail. Second, users could label their occasions as one of a set of common types of drinking occasions in the United Kingdom (eg, “Big night out” and “Quiet night in with family”) (*occasion type*). This approach provides less flexibility in describing the specific characteristics of each occasion but may be less burdensome for users.

The aim of this study was to compare the user engagement, data quality, usability, and acceptability of the 2 methods of collecting information on the contexts of alcohol consumption in the Drink Less app. This work will inform the development of context-based interventions, which are hypothesized to improve intervention effectiveness. The research questions were as follows:

1. Which method of collecting drinking diary data is associated with greater engagement by app users in practice?
2. Which method collects higher quality data on different types of drinking occasions?
3. Which method of collecting drinking diary data performs better on ease of use, interface and satisfaction, and usefulness?
4. How do app users feel about the acceptability of each method of collecting drinking diary data?

## Methods

### Design

This was a randomized mixed-methods usability study. The between-subject factor was the app version that participants were randomized to download and use for 14 days. The study has been registered at OSF [16].

### Drink Less App

The Drink Less app was designed to help people drinking at increasing and higher risk levels to reduce their alcohol consumption, and to achieve short- and long-term improvements in population health across the socioeconomic spectrum [17]. The app is widely used (over 70,000 unique users since its launch in 2016), highly rated by users (4.45/5 stars), and highly visible (included in the top results for “alcohol” searches) on the Apple App Store (only currently available on iOS devices). It includes a drinking diary where users can track the alcoholic products and therefore the units of alcohol that they drink each day.

**Figure 1:**
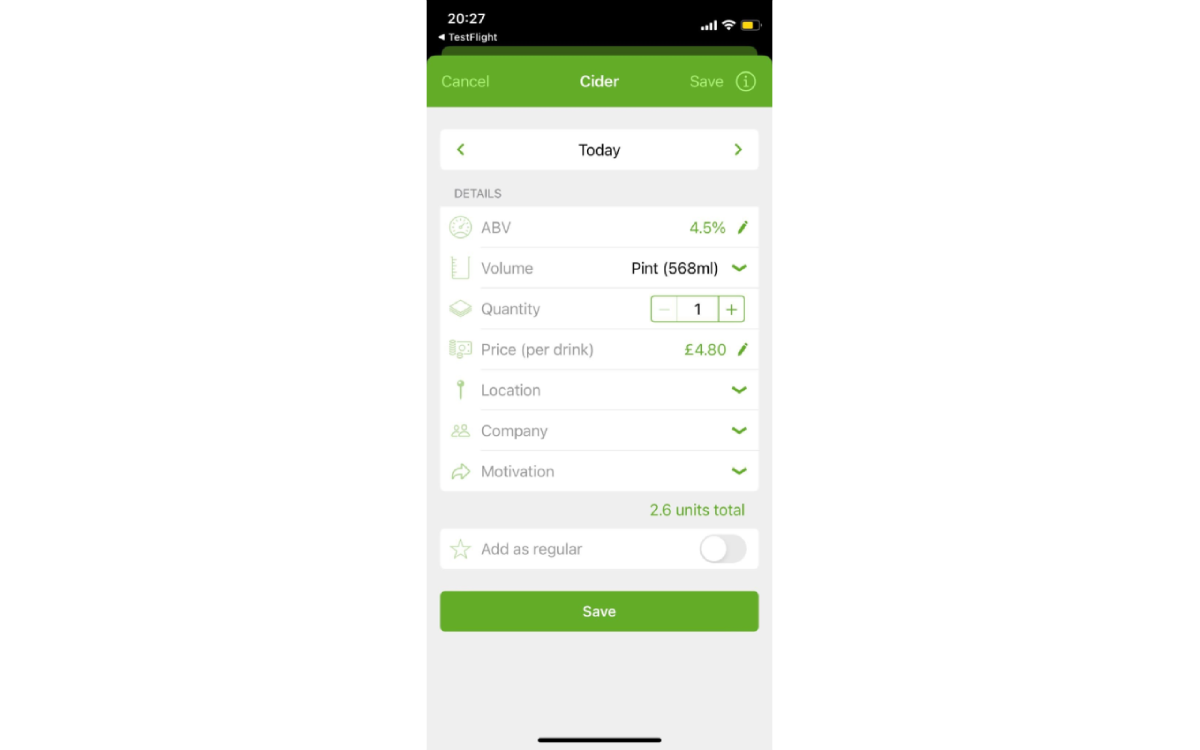
Screenshot of the drinking diary for “tags,” which is a modified version of the Drink Less app that allows users to add 3 characteristic tags to their drinking occasion (location, company, and motivation).

**Figure 2:**
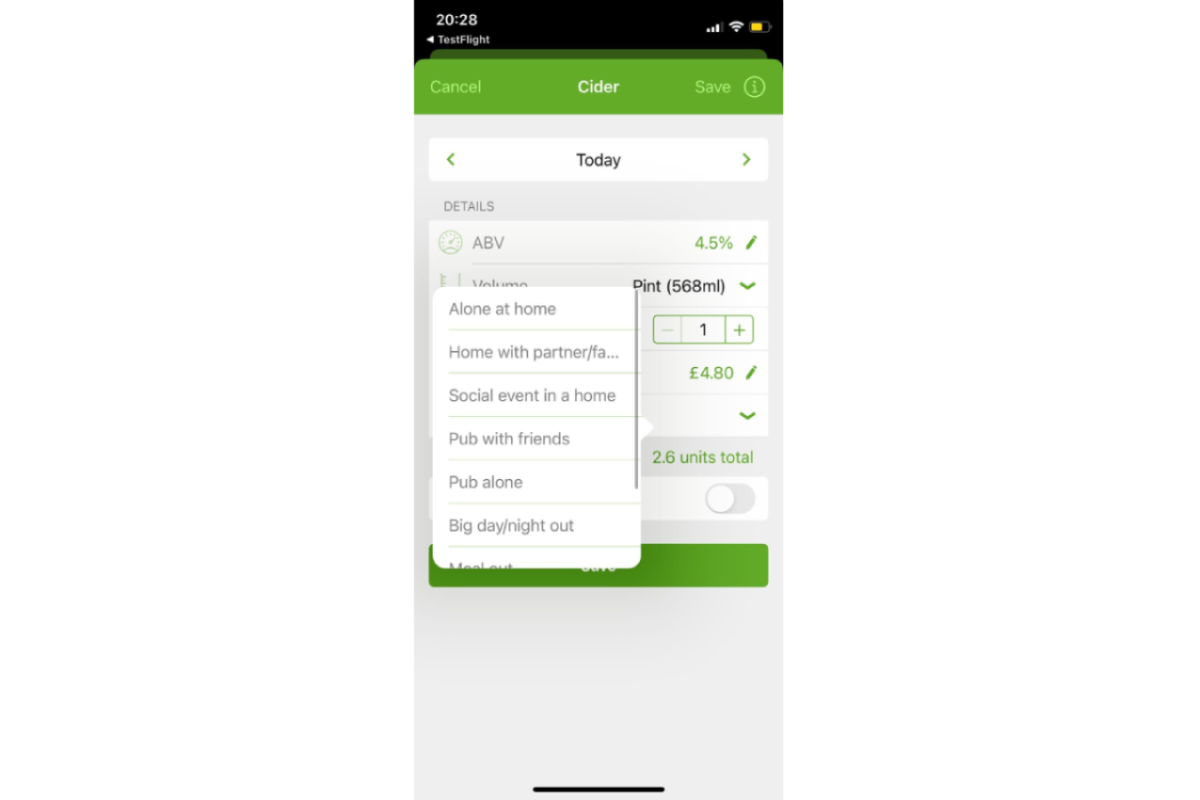
Screenshot of the drinking diary for “occasion type,” which is a modified version of the Drink Less app that allows users to label their drinking occasion as one of a set of common types in the United Kingdom.

We iteratively designed 2 new versions of the app for use in this study, with the aim of balancing their potential for a tailored intervention against participant burden. *Tags* incorporated context tags in 3 categories (ie, location, company, and motivation) into the drinking diary, while *occasion type* used a list of occasion types derived from a recent analysis of drinking occasions in Great Britain [9] (Textbox 1; Figures 1 and 2). For *tags*, the user was required to add a location tag, but could choose not to add company and motivation tags. Only one location, company, and motivation tag could be added to each record. We chose not to make all tags mandatory to avoid additional response burden compared to *occasion type*. For *occasion type*, the user was required to pick an occasion type and could not select multiple options. Participants could enter multiple drinking records per day, and each record had independent context tags or occasion types. Alternatively, they could enter a record to indicate an alcohol-free day.

**Contextual information included in each modified version of the Drink Less app.**

**Tags version – location, company, and motivation tag options**

***Location***

- A home

- Pub, bar, or clubs

- Restaurant or cafe

- Other

***Company***

- Partner

- Friends

- Family

- Alone

***Motivation***

- To fit in

- To relax

- To celebrate

- To cope

- Boredom

**Occasion type version – options to label drinking occasion as one of a set of common types in the United Kingdom**

- Alone at home

- With partner or family at home

- Social event in a home

- Pub with friends

- Pub alone

- Big day or night out

- Meal out

- Out with partner

- Other

### Participants and Screening

The target minimum sample size was 16 participants for each version of the Drink Less app, with 8 per version for qualitative interviews. We selected this sample size as the methodical literature suggests that it is sufficient for detecting usability issues, and it is also in line with a similar study on the usability of the Drinks;Ration app [18,19]. We used the participant panel Prolific to recruit a nonrandom, unstratified convenience sample of 62 participants, who were randomly allocated to use the *tags* (n=31) or *occasion type* (n=31) version of the Drink Less app for 14 days [18]. Recruitment took place in 2022 and 2023. We initially planned to randomly select and invite 8 participants using each version of the app to complete a follow-up interview and planned to recruit more if data or meaning saturation was not met. However, when analyzing data from the last interviews, no new open codes were developed and the final focus group did not change the meaning of any existing codes or themes. As such and in line with previous definitions [20], the research team concluded that theoretical and meaning saturation had been achieved. As such, 16 participants (*tags*, n=8; *occasion type*, n=8) participated in follow-up qualitative interviews.

We screened participants for eligibility using Prolific’s built-in screening questions and a custom screening questionnaire hosted on the survey platform Qualtrics [21]. Eligibility criteria were current UK residents, age ≥18 years, ability to understand written English, access to an iOS device (as the Drink Less app is only available currently on iOS), self-report drinking more than 14 UK units a week (1 unit = 10 mL of ethanol = 25 mL of 37.5% alcohol-by-volume [ABV] spirit), and motivation to cut down drinking by responding “yes” to the question “Do you want to drink less alcohol?” During screening, participants completed the Alcohol Use Disorders Identification Test (AUDIT) and were given a further message informing them that they may be at risk of alcohol dependence and signposting to further support if they scored 20 or more [22]. Both eligible and ineligible participants were reimbursed £1 (US $1.30) for completing the screening survey.

### Procedure

Eligible participants were invited to download and use the app for 14 days. Each participant was independently randomized to either the *tags* or *occasion type* version using a random number generator in Microsoft Excel. We informed participants that we were particularly interested in the drinking diary and asked them to complete this every day. Following 14 days of app use from the date of download, we invited participants to complete the validated mHealth App Usability Questionnaire (MAUQ), which was hosted on the survey platform Qualtrics [23,24]. Participants were eligible to complete the survey if they downloaded the app and reported at least one drinking occasion on one day of the diary. Three participants signed up for the study while participating in Dry January and therefore did not report any drinking occasions. For these people, we restarted the 14 days of app use from the 1st of February to allow them to participate.

In the survey, we included screenshots of the app version that the participant had used (Figures 1 and 2) and asked them to focus on this part when completing the MAUQ. After survey completion, participants were reimbursed a further £20 (US $26.03) irrespective of how many days they completed the diary.

We then invited participants who had used the app and completed the survey to undertake follow-up semistructured interviews to investigate their experiences of the acceptability of the app and drinking diary. The interviews were conducted online by the author MO, a mixed methods researcher. The interviews took 15 minutes on average (between 10 and 26 minutes). Participants received a further £20 (US $26.03) reimbursement following the interview.

### Measures

#### Engagement (Research Question 1)

We collected app usage data during the 14 days after download to assess app engagement and data quality. Measures of engagement were as follows: (1) number of days that the diary is completed (by either reporting a drinking record or reporting an alcohol-free day) in the 14-day period; (2) number of consecutive days that the diary is completed; (3) depth of use (ie, the number of unique available screens viewed); (4) amount of use (ie, total minutes spent on the app over the 14-day period); and (5) frequency of use (number of sessions [a new session is counted after 30 minutes of inactivity] and number of days using the app).

#### Data Quality (Research Question 2)

Measures of data quality were used to assess whether each version collected sufficiently detailed information to inform future intervention development. For the *tags* version, we assessed the proportion of drinking records where participants selected “other” as the location tag and the frequency that participants provided information about each of the areas covered by the tags. For the *occasion type* version, we assessed the proportion of drinking records where participants selected “other” as the occasion type. The selection of “other” may indicate disinterest, inappropriate tags or occasion types, or other problems with providing contextual data.

#### Usability (Research Question 3)

To assess ease of use, interface and satisfaction, and usefulness, participants completed the MAUQ after using the app for 14 days [23]. The MAUQ consists of 3 subscales, which have been described in Multimedia Appendix 1. Participants responded to a series of statements on a Likert scale from 1 (strongly disagree) to 5 (strongly agree) (Cronbach α=.932 [23]).

#### Acceptability (Research Question 4)

Finally, our interviews focused on app users’ feedback on the usability, relevance, and burden of using the app, with a focus on the drinking diary. The interview topic guide (Multimedia Appendix 2) was designed to first measure an individual’s gut feeling about the drinking diary they had used before exploring the 7 component facets within the Theoretical Framework of Acceptability (TFA) [25]: affective attitude, burden, perceived effectiveness, ethicality, intervention coherence, opportunity costs, and self-efficacy. We also included a question on perceived personal relevance [26].

### Analysis

We used descriptive analyses (mean and SD) to compare measures of user engagement, data quality, ease of use, interface and satisfaction, and usefulness between app versions. The study was not powered to support inferential statistics. Stata v17 (StataCorp) was used for quantitative analysis.

Interviews were transcribed verbatim, anonymized, and then uploaded into Nvivo 12 (QSR International) for coding and analysis using both deductive and inductive methods. We developed an initial coding framework using a priori themes (eg, TFA constructs and perceived personal relevance) before reading the transcripts several times and incorporating additional new inductive themes. One researcher (MO) then coded 3 interviews. To ensure the trustworthiness of this analysis, this coding was then checked by a second researcher (LD), and an iterative process of cross-checking coding strategies and data interpretation was carried out to establish a consensus of opinion and develop a revised coding frame. Coding was further refined using an ongoing comparative method, whereby each interpretation and finding was compared with existing findings, as more transcripts were analyzed. Following initial coding, similar responses within each construct were inductively analyzed to generate content themes representing how that construct contributed to reported acceptability. Participant quotes (along with their sex and the version of the app they used) have been provided.

### Ethical Considerations

The human participant research protocol was approved by University College London’s ethics committee (approval number: 23875/004) and University of Sheffield’s ethics committee (approval number: 786), and conforms to the principles embodied in the Declaration of Helsinki. The consent process emphasized that participation was voluntary and that participants could withdraw at any time without penalty. Informed consent was provided by all participants. During the study we used IDs on Prolific to contact participants, and these were deidentified and replaced by an anonymous participant identifier for analysis.

## Results

### Overview

A total of 98 participants were invited to download and use the Drink Less app. Of those, 62 (*tags* version, n=31; *occasion type* version, n=31) chose to participate in the study, completed the MAUQ survey, and were included for quantitative analysis, and 16 (*tags* version, n=8, 26%; *occasion type* version, n=8, 26%) completed the follow-up qualitative interview. Descriptive frequencies of tags and occasion types reported are available in Multimedia Appendix 3.

### Engagement (Research Question 1)

Overall, engagement was high for both groups. For example, the mean number of days participants completed the diary was greater than 12 during the 14-day study period (*tags* version: mean 12.23, SD 2.46 days; *occasion type* version: mean 12.39, SD 2.12 days). However, participant engagement with the app was marginally higher for the *occasion type* version across all measures (Table 1). The standard error for amount of use was higher for the *tags* version owing to 1 user with high total minutes spent on the app. With this user excluded, the mean time spent on the *tags* version was 34.77 (SD 29.50) minutes, while the mean time spent on the *occasion type* version was 48.47 (SD 26.13) minutes.

**Table 1:** Descriptive statistics on engagement, data quality, and mHealth App Usability Questionnaire scores for the “tags” and “occasion type” versions of the Drink Less app used for 14 days.

Variable				Tags version – users add location, company, and motivation tags (n=31), mean (SD)		Occasion type version – users add a label from a set of common types in the United Kingdom (n=31), mean (SD)
**Engagement**						
	Days for completing the diary (any record entered)		12.23 (2.46)		12.39 (2.12)	
	Consecutive days for completing the diary (any record entered)		11.10 (3.55)		11.42 (3.62)	
	Depth of use (number of unique screens viewed)		24.23 (7.47)		26.48 (6.00)	
	Amount of use (minutes spent on the app)		41.64 (48.00)		48.47 (26.13)	
	Frequency of use (number of sessions)		22.58 (12.52)		24.42 (10.26)	
**Data quality**						
	Total number of drinking records		8.74 (6.08)		9.55 (6.02)	
	Proportion of drinking records with “other” as the location tag		0.02 (0.05)		—^a^	
	Proportion of drinking records reporting a company tag		0.61 (0.44)		—	
	Proportion of drinking records reporting a motivation tag		0.56 (0.44)		—	
	Proportion of drinking records with “other” as the occasion type		—		0.00 (0.01)	
**Usability (average MAUQ^b^ score)**				72.39 (8.10)		74.23 (6.76)
	**Ease of use (MAUQ statement)**		22.00 (2.28)		21.97 (2.12)	
		S1. The app was easy to use.	4.52 (0.51)		4.42 (0.56)	
		S2. It was easy for me to learn to use the app.	4.48 (0.63)		4.61 (0.50)	
		S3. The navigation was consistent when moving between screens.	4.52 (0.57)		4.32 (0.79)	
		S4. The interface of the app allowed me to use all the functions (such as entering information, responding to reminders, and viewing information) offered by the app.	4.42 (0.56)		4.32 (0.60)	
		S5. Whenever I made a mistake using the app, I could recover easily and quickly.	4.06 (0.93)		4.29 (0.74)	
	**Interface and satisfaction (MAUQ statement)**		28.90 (3.82)		30.10 (3.20)	
		S6. I like the interface of the app.	3.97 (0.87)		3.84 (0.78)	
		S7. The information in the app was well organized, so I could easily find the information I needed.	4.10 (0.75)		4.10 (0.70)	
		S8. The app adequately acknowledged and provided information to let me know the progress of my action.	4.23 (0.67)		4.35 (0.66)	
		S9. I feel comfortable using this app in social settings.	3.35 (1.20)		4.13 (0.96)	
		S10. The amount of time involved in using this app has been fitting for me.	4.55 (0.51)		4.48 (0.54)	
		S11. I would use this app again.	4.32 (0.79)		4.42 (0.76)	
		S12. Overall, I am satisfied with this app.	4.39 (0.67)		4.58 (0.56)	
	**Usefulness (MAUQ statement)**		21.48 (3.50)		22.16 (2.76)	
		S13. The app would be useful for my health and well-being.	4.23 (0.67)		4.45 (0.72)	
		S14. The app improved my access to health care services.	2.58 (0.89)		2.65 (0.88)	
		S15. The app helped me manage my health effectively.	3.45 (0.93)		3.61 (0.72)	
		S16. This app has all the functions and capabilities I expected it to have.	3.94 (0.68)		4.10 (0.75)	
		S17. I could use the app even when the internet connection was poor or not available.	3.52 (0.77)		3.58 (0.67)	
		S18. This mHealth^c^ app provided an acceptable way to receive health care services, such as accessing educational materials, tracking my own activities, and performing self-assessment.	3.77 (1.02)		3.77 (0.99)	

^a^Not applicable.

^b^MAUQ: mHealth App Usability Questionnaire [23].

^c^mHealth: mobile health.

### Data Quality (Research Question 2)

Data quality was also high for both app versions. For example, the proportion of drinking records with “other” as the location tag in the *tags* version was 0.02 (SD 0.05), while the proportion in the *occasion type* version was 0.00 (SD 0.01). However, a higher proportion of drinking records in the *tags* version (approximately 40%) did not include company and motivation tags (Table 1).

### Usability (Research Question 3)

Usability ratings on the MAUQ were marginally higher overall for the *occasion type* version (*tags* version: mean 72.39, SD 8.10; *occasion type* version: mean 74.23, SD 6.76) (Table 1). The majority of MAUQ item scores differed only slightly between the 2 app versions. Scores were good overall, although users were more critical on the item “The app improved my access to health care services,” which was less relevant for an app tracking alcohol consumption.

### Acceptability (Research Question 4)

Sixteen participants took part in follow-up interviews where they were asked to discuss their experience of using the drinking diary. See Table 2 for participant characteristics (Multimedia Appendix 4 for the full sample), Table 3 for a summary of the themes discussed, and Multimedia Appendix 5 for researcher characteristics and reflexivity.

**Table 2:** Demographic characteristics of usability study participants who completed follow-up interviews after using the “tags” and “occasion type” versions of the Drink Less app for 14 days.

Variable			Tags version – users add location, company, and motivation tags (n=8)		Occasion type version – users add a label from a set of common types in the United Kingdom (n=8)		All users (n=16)
Female sex, n (%)			2 (25)		4 (50)		6 (38)
Age (years), mean (SD)			45.9 (16.2)		44.1 (14.7)		45.0 (15.0)
**Ethnicity, n (%)**							
	White	6 (75)		8 (100)		14 (88)	
	Mixed	2 (25)		0 (0)		2 (13)	
AUDIT^a^ score, mean (SD)			16.5 (6.9)		17 (6.0)		16.75 (6.2)

^a^AUDIT: Alcohol Use Disorders Identification Test.

**Table 3:** Summary of themes discussed by usability study participants who completed follow-up interviews after using the “tags” and “occasion type” versions of the Drink Less app for 14 days.

Theme	Construct definition	Tags version – users add location, company, and motivation tags	Occasion type version – users add a label from a set of common types in the United Kingdom
Affective attitude	How an individual feels about the intervention.	Participants reported liking the drinking diary. One participant reported liking being able to put drinking in context, particularly when she was socializing.	Participants enjoyed using the drinking diary. Participants reported some feelings of guilt or sadness when logging heavy drinking days. Participants reported that they felt more negative affect logging days where they drank at home than days where they were logging drinking occasions where they were socializing with friends. Thought of this as “good” and “bad” reasons for drinking.
Burden	The perceived amount of effort necessary to use the intervention.	Generally considered to be quick and easy to use. Some felt the number of tags was burdensome and felt they should have been optional to add after logging or on a weekly basis.	Generally considered to be quick and easy to use. Context-specific section was generally considered to be quick and easy to use. One participant reported that they found it repetitive.
Ethicality	The extent to which the intervention has a good fit with an individual’s value system.	Drinking diary was not intrusive and was nonjudgemental and inoffensive. Participants felt that it was a good fit and that it supported them to make changes to their own health and reduce their drinking.	Drinking diary was not intrusive and was nonjudgemental and inoffensive. Participants felt that it was a good fit and that it supported them to make changes to their own health and reduce their drinking.
Intervention coherence	The extent to which the participant understands the intervention and how it works.	Generally reported that the drinking diary was straightforward to use. No difficulties with using the context-specific parts of the app.	Generally reported that the drinking diary was intuitive. Few difficulties with using the context-specific parts of the app. One participant reported that one of the occasions was too long to read.
Opportunity costs	The extent to which benefits, profits, or values must be given up to engage with the intervention.	None reported; app was quick to use and participants liked that it was a mobile app.	None reported; app was quick and easy to use. Mobile and discreet nature meant it fit easily into people’s lives.
Perceived effectiveness	The extent to which the intervention is perceived as likely to achieve its aim.	People reported finding the tracking element of the app useful and were surprised by the number of units they drank. Others felt it made them more aware of their drinking but did not help in cutting down. Reflecting on contexts was helpful for some participants. Success in cutting down for some occasions more than others.	People reported finding the tracking element of the app useful and were surprised by the number of units they drank. Others felt it made them more aware of their drinking but did not help in cutting down. Some found the context section useful in reflecting on why they had drunk and felt it particularly highlighted the days where they had drunk mindlessly or were at home or alone. Others found it less useful; they felt they always recorded the same context or could not see where the information was being used.
Perceived personal relevance	The extent to which the intervention is suited to the participant’s individual needs.	Participants mostly felt that the tags fit their drinking occasions. Suggested some additional features, including “habit” and “end of work drinks.”	Participants felt that they were able to map their drinking occasions to the contexts listed. One participant suggested that “work drinks” should be added.
Self-efficacy	The participant’s confidence that they can perform the behaviors required to participate in the intervention.	Generally quite confident but mixed in terms of experience.	Generally confident in the use of the app and had experience in using similar apps for different behaviors. One participant “tested” the app to make sure it was giving the right alcohol-by-volume (ABV) value and reported confidence in it after that.

### Affective Attitude

Participants using both versions of the diary reported enjoying using it.

I did enjoy it, yeah, it looks good and it’s very visual which helps.

Participant #1, male; occasion type version

I liked it and it was good to kind of from my own perspective to look at any kind of patterns or trends in what I was doing.

Participant #2, male; tags version

One participant using the *tags* version said that they liked being able to put their drinking in context, and they mentioned that they liked that they could log a drinking occasion as being a social one.

I think when you’re logging something as like at a pub, at a social that it just feels like kind of, it feels like you're drinking to socialize and not drinking alone in a house. Maybe so. I liked to put it in that context.

Participant #3, female; tags version

Participants using the *occasion type* version reported that they felt more negative affect logging days where they drank at home than days where they were logging drinking occasions where they were socializing with friends. They thought of this as “good” and “bad” reasons for drinking.

If I went out and I was with friends and we all did it at the same, that would seem a lot more…not acceptable, but maybe a bit more normal.

Participant #4, male; occasion type version

…where I did feel a little bit like sad in myself that I just had to put drinking at home alone for three days straight. But I wasn’t just sat at home crying and drinking. I was like reading.

Participant #8, female; occasion type version

Other participants using the *occasion type* version reported feelings of guilt or sadness when logging heavy drinking days.

…it came up heavy drinking I think it actually described it as, and I’d only had 4 pints it made me feel a little bit guilty to be honest.

Participant #1, male; occasion type version

### Burden

Participants reported that the drinking diary was quick and easy to use, with most people in both versions saying that it took them 2 minutes or less to log their drinks. Most felt that the balance was right between entering enough information to get informative feedback and being quick to use.

I tended to pick it up, go in, couple of clicks and I’ve done it.

Participant #5, female; occasion type version

I either tend to drink at home or when we go out as a couple for a meal. So yeah, that that all seemed fairly easy to put in.

Participant #6, male; tags version

It like covered all the sort of situations you would probably have a drink in, whether it was on your own or in the house or whatever. And so yeah, that was good. And it was easy to use.

Participant #7, female; occasion type version

Some participants using the *tags* version reported finding that the tags could be burdensome at times and suggested that they could be optional addons after logging a drink or could be done on a weekly, rather than daily, basis. In the *occasion type* version, some found that recording the context could feel repetitive.

Erm there was quite a lot on there. So you just tended to just pick the location because it was just a bit a bit quicker maybe.

Participant #10, male; tags version

It’s always the same context, so I found that a bit repetitive. But I can see the point of it.

Participant #5, female; occasion type version

### Ethicality

Participants using both versions felt that the app was not intrusive and was nonjudgemental and inoffensive.

It’s not like in any way trying to make you feel bad or trying to make you feel good. It’s just giving you cold, cold hard facts.

Participant #4, male; occasion type version

Participants felt that it was a good fit with their values and that it supported them to make changes to their own health and reduce their drinking.

I was never conscious that my drinking was impacting on my health, but logically it was going to at some point, so yes, it fits in with my values that I'm responsible for this and I had to do something about it.

Participant #5, female; occasion type version

One participant felt that the goal of the app was not aligned with their own goal of reduction rather than temperance.

It’s clearly color-coded, so obviously if you’ve got a week that went above the recommended limit, it turns it red. But if you’re below, it’s amber. So it does sort of feel like it’s a zero alcohol app, rather than a reduce alcohol app.

Participant #9, male; occasion type version

### Intervention Coherence

Participants using both versions of the app generally reported that the drinking diary was straightforward to use. They did not report any difficulties with using the context-specific parts of the app.

One participant using the *occasion type* version reported that one of the occasions was too long to read.

It’s not completely clear because the I think it was too long and it’s sort of abbreviated. So it’s like home with partner/fa, which I think is family? I’m not sure. But yeah, the others seemed fine.

Participant #9, male; occasion type version

### Opportunity Costs

Completing the drinking diary was not seen to interfere with anything else important to participants. It was felt that due to its speed and the mode of delivery as an app, the drinking diary fit into their lives easily.

I used to fill it in no matter where I was, if I was on a bus, if I was in work.

Participant #3, female; tags version

If you’re in a pub, it was easy to do like if my mate was going for a pee or I don’t know. People sit on the smartphones for a bit anyway. You’ll have to message someone so. It didn’t feel like it was a chore to do.

Participant #9, male; occasion type version

### Perceived Effectiveness

Participants using both versions of the drinking diary felt that the drinking diary was useful for them, and they reported being surprised by how many units they were drinking and reported using the app to reduce their consumption.

Absolutely like 100% [it helped reduce consumption] like I had no idea that I was drinking so much and how bad that is.

Participant #7, female; occasion type version

I liked knowing my alcohol-free days that I had, it kind of encouraged me to make sure, I had days, rather than maybe having one drink and uhm and having to log that.

Participant #3, female; tags version

It has helped me in January really, some days when I thought I’ll just have a cheeky pint and I think no I want I wanna keep that day green do you know what I mean?

Participant #1, male; occasion type version

Others reported that the app made them more aware of how much they were drinking but did not necessarily impact their consumption.

I think I was more conscious during that period of, like, oh, I’d drank a lot this week, but I wasn't necessarily like motivated to drink less. I was more just aware if that makes sense.

Participant #11, male; tags version

Some found the context section useful in reflecting on why they had a drink.

I felt that was either kind of giving you an indication of the pattern whether you were drinking alone, more so than actually using it for socializing.

Participant #3, female; tags version

I think it’s something I was already thinking about is just because there's a football game on, you don't have to have a drink for that.

Participant #2, male; tags version

A couple of drinks in the house is one thing, but when you’re getting to like 8 or 9. I was like, wow, it is that it’s escalating a little bit…so yeah, certainly helps.

Participant #4, male; occasion type version

Participants reported that the drinking diary was differentially effective for different drinking contexts. People used the app mainly to encourage themselves to have more alcohol-free days or to cut out drinking at home or when they were alone.

I would like see that when you're out and having a drink like in the pub or whatever, I think that's fine, but two of mine were just in the house and doing nothing and just drinking then, which when you sort of see it written down you're like well, that's actually just pointless almost. Why am I doing that? So yeah, I think it that helped me a bit.

Participant #7, female; occasion type version

When I’m out, I can actually ignore things a lot easier. If I’m at home and then I drink then if you’re logging it in something, then you’re a lot more conscious of it. So it would it made me drink less when I was at home.

Participant #11, male; tags version

Others using the *occasion type* version found it less useful, and they felt they always recorded the same context or could not see where the information was being used.

It’s always the same context. You know, I walked through to the kitchen and my husband’s there and we get the meal ready. That’s always the context.

Participant #5, female; occasion type version

### Perceived Personal Relevance

Participants felt that they were able to map their drinking occasions to the contexts listed, though some participants mentioned some additional options that they thought would be a good fit. In the *tags* version, this included “habit” as a motivator of drinking and considering temporal points such as the end of the work day. In the *occasion type* version, 1 participant suggested adding work drinks.

I could definitely easily put in each like put which one was right for the event that happened.

Participant #8, female; occasion type version

Well, where you were drinking or who you were drinking with wasn’t it? The reason why you thought you were drinking? No, I think that covered it, you could put yourself into the boxes from it.

Participant #12, female; tags version

I think, within the context, I think it’s just missing habit. Perhaps, I think sometimes I just drink cause it’s a it’s what I do when I get home when I start cooking dinner and there wasn’t a sort of specific context that seemed to cover that.

Participant #6, male; tags version

### Self-Efficacy

Participants using both versions reported confidence in using the app to track their drinking due to their familiarity with technology and other apps. One participant “tested” the app to make sure it was giving the right ABV values and reported confidence in it after that.

I haven’t used anything like that specifically, but I you know I use other apps like Strava and stuff so I’m used to finding my way around things.

Participant #6, male; tags version

I did check the ABV on what I was drinking and your values were, I think, within .1 of a percent…it was close, close enough. So yes, I trusted it.

Participant #5, female; occasion type version

Participants in both conditions reported contextual factors, which they thought impacted their ability to change their behavior.

It’s a difficult time of year as always when you're looking at the middle of December.

Participant #13, male; occasion type version

It came at a good time actually because of like the dry January thing and lots of people have been talking about it. And I… although I didn’t do dry January, I did try and cut down and that helped.

Participant #14, male; tags version

## Discussion

### Principal Findings

We found that both modified versions of the Drink Less app were implemented successfully, with similarly high levels of engagement, usability, and acceptability. Data quality was good, with minimal data recorded as “other,” and participants recorded sufficient data on their drinking contexts to suggest that an adapted version of Drink Less could be used as the basis for context-specific interventions. These positive findings align with qualitative evidence that in the context of smoking cessation apps, users most often mention tracking progress, smoking patterns, and psychological triggers as important app functions [27]. In our qualitative interviews, users gave some suggestions for developing each version further, such as the inclusion of “work drinks” in both versions and the inclusion of “habit” as a motivation in the *tags* version, which the research team will consider in future work.

The comparative quantitative measures showed no substantive evidence of a difference between the *tags* and *occasion type* versions of the app, suggesting that either is viable for future use. However, a substantial minority (approximately 40%) of *tags* drinking records did not include the optional motivation and company tags. This may reduce the potential to offer targeted interventions when using this approach and therefore limits the value of the more detailed and more burdensome approach. In our study, the motivation and company tags were the only optional contextual information. However, for digital health interventions released to the general public, it is important to minimize participant burden, as additional requirements for users are associated with reduced long-term intervention use [28,29]. This was demonstrated in the qualitative interviews where participants spoke about the balance of measuring detailed enough information to get meaningful information while ensuring the app did not become too burdensome for users. The majority of individuals interviewed felt that both versions of the app were easy to use. However, 2 individuals found that the tags were detrimental to their user experience. Part of this may be because they could not see where the information was being used, as the information in the current version of the app was not carried forward into the intervention components.

### Implications and Future Research

In terms of the next steps for the Drink Less app, given that the ratings were similar across both versions of the context-specific drinking diary, we will take forward the less burdensome version for future development, which will include developing context-specific advice within the intervention components in collaboration with experts in behavior change and potential app users [30]. This will enable us to test the efficacy of context-specific advice in future research, which could have significant implications for intervention development. Another factor to consider when continuing development of the context-specific iteration of the app is unintended consequences. In qualitative interviews, participants in both conditions spoke about how the app helped them to reduce their alcohol consumption more in some drinking contexts than others. Participants reported that they used the app mostly to cut out lone or home drinking occasions, which are common in Great Britain [31], but that they were less likely to reduce consumption in social settings. Some participants spoke about the social benefits of alcohol consumption and suggested that they felt less negative affect recording social drinking contexts out of the home than they did with lone or home drinking. This raises 2 issues that should be considered in the application of context-specific intervention work for alcohol consumption. First, it supports arguments that some types of drinking may be more amenable to change than others and that tailoring interventions to these types, as well as those associated with the highest volumes of alcohol consumption, may be an effective intervention strategy [3,7,32]. Second, there may be unintended consequences whereby drinkers feel less guilt or negative affect when logging heavier social drinking occasions [33].

### Strengths and Limitations

Despite the increasing attention paid to drinking contexts by public health researchers, this study is one of the first to consider the practical aspects of developing context-based interventions. The study met recruitment targets as reported in our preregistration. Participants were engaged with and positive about their experiences using the adapted versions of the Drink Less app. Participants felt that the app used nonjudgemental language and was easy to use in their daily lives, which may encourage more honest reporting in the drinking diary [18,34].

A limitation of this study is the use of a convenience sample, which consisted of mostly white people who were older than 30 years. The sample may also have high levels of digital literacy compared to the general population. This approach was suitable for the aims of this usability study, but the findings may not be generalizable, and the modified versions of the Drink Less app would require further evaluation before implementation. We were unable to explore differences in engagement, usability, and acceptability across population subgroups, including those with lower or higher alcohol consumption, due to our small convenience sample. Second, we did not compare the participant burden of the original app with our modified versions. Although we found evidence of acceptability for both modified versions, future evaluation work should weigh the hypothesized improved effectiveness against increased burden.

### Conclusion

This study demonstrates the feasibility of collecting information on drinking contexts within an alcohol reduction app using 2 different methods. Both the *tags* and *occasion type* versions had high levels of engagement, data quality, usability, and acceptability. Where possible, feedback from qualitative interviews will be incorporated into further work developing context-specific interventions within the Drink Less app, such as including an “after work drinks” occasion type. A more general consideration from the qualitative interviews is to ensure that context-specific interventions are designed to minimize the risk of unintended positive reinforcement of drinking occasions that are seen as sociable by users.

## Appendix

Multimedia Appendix 1 mHealth App Usability Questionnaire statements.

Multimedia Appendix 2 Interview schedule for usability study participants who completed follow-up interviews after using a modified version of the Drink Less app for 14 days.

Multimedia Appendix 3 Descriptive frequencies of contextual information reported for 2 modified versions of the Drink Less app used for 14 days by UK residents drinking at increasing and higher risk levels.

Multimedia Appendix 4 Demographic characteristics of all usability study participants who used a modified version of the Drink Less app for 14 days.

Multimedia Appendix 5 Characteristics and reflexivity of researchers involved in conducting and analyzing qualitative interviews.

## Abbreviations

ABV: alcohol-by-volume
MAUQ: mHealth App Usability Questionnaire
TFA: Theoretical Framework of Acceptability

## Data Availability

The datasets generated during or analyzed during this study are deposited in the ORDA repository [35]. The datasets are available to researchers on request in line with study ethics approval.
