# Supplementary material for: Optimizing the Measurement of Information on the Context of Alcohol Consumption Within the Drink Less App Among People Drinking at Increasing and Higher Risk Levels: Mixed-Methods Usability Study
Source: JMIR Form Res. 2024 Oct 24;8:e50131. doi: 10.2196/50131 (PMC11544327; doi:10.2196/50131)
Supplement: Multimedia Appendix 1 [file formative_v8i1e50131_app1.docx]

**Multimedia Appendix 1.** mHealth App Usability Questionnaire statements.

**Ease of use**

S1. The app was easy to use.

S2. It was easy for me to learn to use the app.

S3. The navigation was consistent when moving between screens.

S4. The interface of the app allowed me to use all the functions (such as entering information, responding to reminders, viewing information) offered by the app.

S5. Whenever I made a mistake using the app, I could recover easily and quickly.

**Interface and satisfaction**

S6. I like the interface of the app.

S7. The information in the app was well organized, so I could easily find the information I needed.

S8. The app adequately acknowledged and provided information to let me know the progress of my action.

S9. I feel comfortable using this app in social settings.

S10. The amount of time involved in using this app has been fitting for me.

S11. I would use this app again.

S12. Overall, I am satisfied with this app.

**Usefulness**

S13. The app would be useful for my health and well-being.

S14. The app improved my access to health care services.

S15. The app helped me manage my health effectively.

S16. This app has all the functions and capabilities I expected it to have.

S17. I could use the app even when the Internet connection was poor or not available.

S18. This mHealth app provided an acceptable way to receive health care services, such as accessing educational materials, tracking my own activities, and performing self-assessment.
