# Supplementary material for: Optimizing the Measurement of Information on the Context of Alcohol Consumption Within the Drink Less App Among People Drinking at Increasing and Higher Risk Levels: Mixed-Methods Usability Study
Source: JMIR Form Res. 2024 Oct 24;8:e50131. doi: 10.2196/50131 (PMC11544327; doi:10.2196/50131)
Supplement: Multimedia Appendix 2 [file formative_v8i1e50131_app2.docx]

**Multimedia Appendix 2.** Interview schedule for usability study participants who completed follow-up interviews after using a modified version of the Drink Less app for 14 days.

Introduction: Throughout this interview we will be asking you about your experience of using the Drink Less app. We are particularly interested in your experience of using the drinking diary. This is the section where you could record drinks you had consumed alongside information about the contexts in which you consumed alcohol.

1. How would you rate the drinking diary on a scale of 1 to 5 stars? And can you tell me why? [Global acceptability]

2. How much did you like the drinking diary? [Affective Attitude]

Which features did you like? Did you like the drinking contexts?

3. Do you believe the drinking diary was suited to your individual needs? [Perceived Personal Relevance]

Could you find the drinks you wanted to? Did the drinking contexts capture your occasions or did you find yourself using the ‘other’ a lot?

4. Do you believe that using the drinking diary helped you to drink less? [Perceived personal usefulness/ perceived effectiveness]

Did recording/thinking about the contexts in which you were drinking help with that?

5. Did you find the drinking diary time-consuming? [Burden]

6. Did you have any other difficulties using it? [Burden]

7. Do you think the drinking diary fits with your values? [Ethicality]

Did anything about it offend you? Do you think anyone could use it?

8. Did using the drinking diary interfere with anything else important to you? [Opportunity costs]

Did you feel you were able to fit it in to your life easily?

9. Did you feel confident in using the drinking diary? [Self-efficacy]

10. Was it clear to you how to use the drinking diary and how it worked? [Intervention coherence]

11. Do you have any other comments you would like to make?
