# Supplementary material for: Optimizing the Measurement of Information on the Context of Alcohol Consumption Within the Drink Less App Among People Drinking at Increasing and Higher Risk Levels: Mixed-Methods Usability Study
Source: JMIR Form Res. 2024 Oct 24;8:e50131. doi: 10.2196/50131 (PMC11544327; doi:10.2196/50131)
Supplement: Multimedia Appendix 3 [file formative_v8i1e50131_app3.docx]

**Multimedia Appendix 3.** Descriptive frequencies of contextual information reported for 2 modified versions of the Drink Less app used for 14 days by UK residents drinking at increasing and higher risk levels.

|  | Number of times reported (%) |
| --- | --- |
| **Tags version – users add location, company and motivation tags** |  |
| ***Location*** |  |
| A home | 169 (62.4) |
| Pub, bar, or clubs | 73 (26.9) |
| Restaurant/café | 18 (6.6) |
| Other | 11 (4.1) |
| *Total* | 271 |
| ***Company*** |  |
| Partner | 62 (35.4) |
| Friends | 26 (14.9) |
| Family | 43 (24.6) |
| Alone | 44 (25.1) |
| *Total* | 175 |
| ***Motivation*** |  |
| To fit in | 4 (2.6) |
| To relax | 116 (76.8) |
| To celebrate | 20 (13.2) |
| To cope | 4 (2.6) |
| Boredom | 7 (4.6) |
| *Total* | 151 |
| **Occasion types – users add a label from a set of common types in the UK** |  |
| Alone at home | 29 (9.8) |
| With partner/family at home | 139 (47.0) |
| Social event in a home | 21 (7.1) |
| Pub with friends | 57 (19.3) |
| Pub alone | 7 (2.4) |
| Big day/ night out | 6 (2.0) |
| Meal out | 23 (7.8) |
| Out with Partner | 13 (4.4) |
| Other | 1 (0.3) |
| ***Total*** | 296 |
