# Supplementary material for: Optimizing the Measurement of Information on the Context of Alcohol Consumption Within the Drink Less App Among People Drinking at Increasing and Higher Risk Levels: Mixed-Methods Usability Study
Source: JMIR Form Res. 2024 Oct 24;8:e50131. doi: 10.2196/50131 (PMC11544327; doi:10.2196/50131)
Supplement: Multimedia Appendix 4 [file formative_v8i1e50131_app4.docx]

**Multimedia Appendix 4.** Demographic characteristics of all usability study participants who used a modified version of the Drink Less app for 14 days.

|  | **Tags version - users add location, company and motivation tags (n = 31)** | **Occasion type - users add a label from a set of common types in the UK (n = 31)** | **All (n = 62)** |
| --- | --- | --- | --- |
| **Age** |  |  |  |
| 18-24 | 4 | 0 | 4 |
| 25-34 | 6 | 6 | 12 |
| 35-54 | 16 | 18 | 34 |
| 55+ | 5 | 7 | 12 |
| **AUDIT score [m(sd)]** | 15.5 (6.1) | 17.9 (7.2) | 16.7 (6.7) |
