# Supplementary material for: Optimizing the Measurement of Information on the Context of Alcohol Consumption Within the Drink Less App Among People Drinking at Increasing and Higher Risk Levels: Mixed-Methods Usability Study
Source: JMIR Form Res. 2024 Oct 24;8:e50131. doi: 10.2196/50131 (PMC11544327; doi:10.2196/50131)
Supplement: Multimedia Appendix 5 [file formative_v8i1e50131_app5.docx]

**Multimedia Appendix 5.** Characteristics and reflexivity of researchers involved in conducting and analyzing qualitative interviews.

Personal Characteristics of Researchers: MO is a female senior research fellow at UCL and has a PhD in Health Psychology. She is a mixed-methods researcher and has previously conducted semi-structured interviews and thematic analysis. She conducted the interviews.

LD is a female PhD researcher in Developmental Cognitive Neuroscience. She has experience in conducting interviews remotely and has undertaken training sessions in qualitative research. LD contributed to the analysis.

Relationship with Participants: Participants had not met the researchers before the focus groups but had exchanged emails regarding logistics of interviews. The goals of the specific research were explained to participants at the start of the interview.

Data Collection: That the focus was on an alcohol reduction app may indicate to participants that we have opinions on alcohol consumption and drinking practices. We are aware that this could create a perceived imbalance of power. Participants might feel pressured to hide how much or the ways in which they drink, or to present a version of themselves that is more concerned about drinking less. We aimed to create a non-judgemental and safe environment in which participants felt able to express their views honestly and openly (e.g. MO shared that they drink alcohol).

Analysis: One of MO’s primary research interests is the development of context-specific interventions for alcohol reduction and so a potential bias may be in interpreting transcripts to support the idea that measuring contexts in the app was perceived as being helpful. Whilst complete detachment on the part of the researcher in relation to data collection and analysis is unattainable, steps were taken to minimise bias. Both MO and LD (who is not focused on drinking contexts or alcohol interventions) coded three interviews before meeting to discuss the codes. There was a high level of agreement across researchers. MO then continued coding the interviews.
